# MONTUR project: Dataset for understanding and forecasting tourist flows

**DOI:** 10.1371/journal.pone.0335190

**Published:** 2025-10-27

**Authors:** Marco Alderighi, Tiziana Ciano, Massimiliano Ferrara, Domenico Santoro

**Affiliations:** 1 Department of Economics, Management and Quantitative Methods, University of Milan, Milano, Italy; 2 Department of Economics and Political Sciences, University of Aosta Valley, Aosta, Italy; 3 Department of Law, Economics and Human Sciences, University Mediterranea of Reggio Calabria, Reggio Calabria, Italy; 4 Department of Economics, Statistics and Business, Faculty of Technological and Innovation Sciences, Universitas Mercatorum, Roma, Italy; Northeastern University (Shenyang China), CHINA

## Abstract

This study presents an advanced system for monitoring and forecasting tourist flows in the Aosta Valley using distributed sensor technologies, cameras, and machine learning algorithms. This innovative system is designed to provide real-time data on arrivals and presences throughout the region, helping to manage traffic and tourism resources more effectively. The research analyzes data collected from portals equipped for traffic detection. Through a multi-phase approach, the project integrates and analyzes over 41 million vehicle passages to support informed decisions for regional economic and social policies. Furthermore, computational processes were conducted to optimize the analysis of the vehicle flow, reducing the dataset and focusing on checkpoints and vehicle categories. This type of time series revealed high stationarity, allowing the use of the eXtreme Gradient Boosting (XGBoost) algorithm for more accurate forecasts than Deep Learning models and other Machine Learning algorithms, such as those highlighted in terms of MAE and MSE. The results represent a significant step forward in managing tourist flows and improving the Aosta Valley’s operational efficiency and visitor experience.

## Introduction

In recent years, thanks to the improvement in living standards, tourism has grown significantly in importance as a means of leisure and lifestyle for people worldwide. Accurate forecasting of the number of visitors is necessary to plan tourist destinations, help hotels and restaurants manage resources, staffing, and inventory effectively, and, more generally, support all operators in the tourism sector in making informed decisions about marketing, pricing, and service delivery [1]. Long-term and short-term projections are the two categories into which tourism flow forecasts can be separated. Both have significant implications, and tourism industry experts can benefit from identifying an accurate trend [2,3], especially when it comes to issues such as the best use of available resources [4]. From the viewpoint of the impact of tourism flow forecasting on tourist destinations studies on rural areas, such as the analyses of Jinshitan [5,6], emphasize the morphological and socioeconomic transformation of tourism-oriented settlements, showing how tourism drives shifts in land use, employment structures, and spatial reconstruction. These works demonstrate the dual impact of tourism: fostering economic growth while reshaping settlement structures, with policy and environmental protection emerging as key factors for sustainable development. Complementing this perspective, research on low-carbon tourism in characteristic towns [7] highlights the importance of striking a balance between emission reduction and cultural and economic vitality. By applying multi-agent models, system dynamics, and mediation analysis, the study captures the interplay between tourists, residents, and enterprises, stressing the centrality of stakeholder behavior and policy support in driving effective low-carbon transitions. Other contributions adopt a broader urban and environmental perspective. The investigation of commuting-related *CO*_2_ emissions in Jinan [8] employs Machine Learning to uncover non-linear relationships between the built environment and emissions, offering planning insights that go beyond traditional linear models. Similarly, the metropolitan-level analysis of carbon balance in China [9] develops a dynamic–static classification framework to evaluate the interaction between economic and ecological factors, providing differentiated strategies for regional low-carbon transformation. The interconnectedness of tourism development, spatial restructuring, and carbon governance requires integrated strategies that balance economic growth, environmental sustainability, and social well-being. The forecast of the number of visitors in tourist areas is influenced by a variety of variables, such as temperature [10], climate [11], and weather [12]. Due to the inevitable imbalance in visitor flows caused by time and weather constraints, tourism is inherently seasonal [13,14]. In the existing literature, a variety of techniques have been used to forecast tourism flows, such as causal econometric models [15,16], non-causal time series models [3,17] and recent advances in machine learning and artificial intelligence offer new approaches to predict tourist flows with greater accuracy [18–22]. [23] discuss analyzing and forecasting tourist flows in Uzbekistan using econometric modeling. They focus on seasonal characteristics and the development of an additive model. The model forecasts future numbers of tourists with a deviation of 20%. Indeed, today, many destinations utilize advanced technologies, including big data, distributed sensors, and machine learning algorithms, to monitor and forecast tourist flows. These tools enable the collection of real-time data on visitor movements, allowing authorities and industry operators to manage resources more effectively and plan for tourist reception. In this regard, the University of Aosta Valley was a partner in a project (*MONTUR: Real-time monitoring and forecasting of tourist flows in Aosta Valley through distributed sensors and machine learning and big data tools*) in response to the “R&D Aggregations” call promoted by the Department of Economic Development, Training, and Labor of the Autonomous Region of Aosta Valley, which aims to create a monitoring and forecasting system (SMP) of tourist flows in Aosta Valley, providing real-time information and accurate forecasts on arrivals and presences in the Aosta Valley territory. Existing approaches to tourism flow forecasting present several limitations. Traditional statistical models, such as ARIMA or SARIMA, often assume linear relationships and therefore struggle to capture nonlinear dynamics, strong seasonality, and the influence of external factors, including geopolitical events or weather variability. More recent Artificial Intelligence-based models, as Deep Neural Networks, can address some of these aspects but are prone to overfitting, require extensive computational resources, and depend on the availability of large volumes of high-quality data, which are not always accessible in regional contexts. By contrast, the methodology proposed in this study seeks to overcome these issues by leveraging traffic-derived features as proxies for tourism flows and integrating them into machine learning models to balance predictive performance and interpretability. Utilizing cameras and sensors (already present in the area) with absolute respect for privacy enables us to obtain anonymized data flows with a density of information that has a much broader scope than the official statistics currently available. Since many factors could potentially influence tourist arrivals and there is a lag between events and their effects on arrivals [3,20], accurate forecasting of tourist arrivals is essential. Still, it represents a well-known challenge for scholars and destination managers [24]. Therefore, using machine learning and big data tools, it is possible to measure the number of visitors (people counting) and classify visitors based on their characteristics and habits (length of stay and destinations). Combining this information with weather and historical data accurately forecasts arrivals and presence in the Aosta Valley area. Introducing new devices improves monitoring quality and accuracy in forecasting, even at the level of a single valley. The Aosta Valley is not equipped with a real-time tourist flow measurement system, nor with a forecasting system for the same, hence the idea to create a product to bring this knowledge. The data provided will allow any stakeholder to make the most profit regarding economic and environmental policies, or for social and public purposes. The Project does not involve using technologies that can modify or alter the surrounding environment. The economic impact we foresee is more than positive; precise data enable a careful analysis of demand from the end consumer, allowing for more targeted decisions on economic policies to be made. A social aspect is also highlighted through collaboration, both during and after the project, with people in the Aosta Valley area. Therefore, the Montur project has developed a demonstrator for a system that monitors and forecasts tourist flows in the Aosta Valley. The system forecasts vehicle transits through 14 different gates in the Aosta Valley based on real-time information. For each gate, the aggregate passage data and its forecast are provided, and the breakdown is made by 7 types of users (including 2 categories of tourists). The model provides hourly and daily short-, medium-, and long-term forecasts.

Despite the increasing interest in tourism flow forecasting, existing approaches face several limitations. Many studies rely on data aggregated at a daily or monthly scale, which may fail to capture short-term dynamics relevant for operational planning. Other studies utilize case-specific datasets that are challenging to replicate in different regional contexts, thereby limiting the generalizability of their findings. In addition, the preprocessing steps required to make raw traffic data suitable for Machine Learning applications are often under-documented, leaving a gap between data collection and model deployment. This study addresses these shortcomings by:

- Providing a novel dataset of hourly tourist flows in the Aosta Valley region (Italy), clustered by vehicle type and access gate;
- Obtaining new, leaner, and computationally easier-to-handle datasets, compared to the original one;
- Evaluating the turnout (in the region) in specific periods of the year. The acquisition methods can be replicated for other regions (Italian and non-Italian), leaving the vehicle clustering unchanged;
- Demonstrating the applicability of these data to Machine Learning algorithms for accurate forecasting.

## Materials and methods

**Fig 1:**
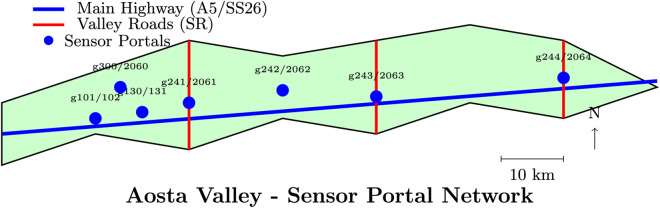
Geographic distribution of sensor portals across the Aosta Valley region. Blue circles indicate the 14 strategic monitoring points positioned at valley entrances and major transportation corridors. The network captures traffic flows from main highways (A5, SS26) and secondary valley roads leading to tourist destinations.

The characteristics of the traffic network of the Aosta Valley, characterized by high-capacity roads on the valley floor (motorways and state roads) on which the roads of the lateral valleys converge, combined with the characteristics of the demand, which presents notable peaks due to tourist flows, generate temporary congestion phenomena in specific and repetitive points. At particular moments, for example, at the end of a holiday day, peaks in demand form, which quickly generate significant flows of traffic, creating critical issues on the network’s weak points, typically those interconnecting with the motorway. Furthermore, the road network of the Valley appears to be strongly influenced by specific weather conditions (such as snow and ice), the presence of roadworks, or serious accidents. Therefore, Montur’s objective is to establish the foundations for creating a capable system that continuously monitors the road network, detects anomalies, and promptly implements interventions to mitigate the effects of anomalies. The monitoring must constantly make the information collected on the road network available. This information is used for timely and efficient traffic management, while also being disseminated to other operators and the public. The information is collected in digital form by an infrastructure made up of “portals” equipped with optical devices and software systems for the initial local processing of the data. The portals detect the data necessary for reporting traffic measurement parameters (for example, the count and average speed of vehicles) and analyze the images for the optical recognition of other characteristic elements of the vehicles in transit. These are positioned on regional roads to guarantee the analysis of traffic at the entrance to as many side valleys as possible. Table 1 provides specific information on the location of the “portals”, while Fig 1 shows their geographical distribution within the Aosta Valley region. The “portals” positioned at these strategic points enable us to capture the incoming/outgoing traffic for the entire region. Their position will enable them to capture not only the local traffic but also consider the vehicles moving towards France, Switzerland, and Piedmont.

**Table 1:** Specific characteristics of the portal.

Gate	Description	Direction	ID Street	City	Coordinates
g101	Fondovalle - Statale	Aosta	SS26	Pont-Saint-Martin	(45.5886, 7.801125)
g102	Fondovalle - Statale	Torino	SS26	Pont-Saint-Martin	(45.5886, 7.801125)
g130	Fondovalle - Autostrada	Aosta	A5	Pont-Saint-Martin	(45.593153, 7.7882)
g131	Fondovalle - Autostrada	Torino	A5	Pont-Saint-Martin	(45.593153, 7.7882)
g241	Valle d’Ayas	Ayas	SR45	Verrès	(45.671397, 7.694394)
g242	Valle di Valtournenche	Valtournenche	SR46	Antey-Saint-Andrè	(45.769131, 7.599786)
g243	Valle di Cogne	Cogne	SR47	Aymavilles	(45.700783, 7.248894)
g244	Val di Rhêmes e Valsavarenche	Rhêmes - Valsavarenche	SR23	Villeneuve	(45.696744, 7.194747)
g300	Valle del Lys	Gressoney	SR44	Pont-Saint-Martin	(45.597742, 7.802783)
g2060	Valle del Lys	Fondovalle	SR44	Pont-Saint-Martin	(45.597742, 7.802783)
g2061	Valle d’Ayas	Fondovalle	SR45	Verrès	(45.671397, 7.694394)
g2062	Valle di Valtournenche	Fondovalle	SR46	Antey-Saint-Andrè	(45.769131, 7.599786)
g2063	Valle di Cogne	Fondovalle	SR47	Aymavilles	(45.700783, 7.248894)
g2064	Val di Rhêmes e Valsavarenche	Fondovalle	SR23	Villeneuve	(45.696744, 7.194747)

Each portal will be equipped with software for transmitting the detected data, including the possibility of setting threshold values outside of which alarm signals will be automatically generated. The data collected through the portals is processed centrally to derive traffic parameters, which can be reported on a dashboard that visually represents the state of the road system. The data flow from the territorial portals is stored in the database appropriately set up in the central system. The central system comprises the processing unit for real-time traffic data, the central system for processing transit images, the server for collecting and disseminating real-time video surveillance images of traffic conditions, and the related connections and interfaces to other systems. Most portals have the same components: a T-EXSPEED v.2.0 and a T-ID unit capable of providing vehicle transits in both directions. This allows for redundant automatic recognition through license plate reading for almost all vehicles in transit, except for motorcycles (not equipped with a front license plate), which statistically represent a negligible percentage of traffic. Both devices use the proprietary license plate recognition program developed by KRIA s.r.l. (Knowledge Research in Imaging Applications). It has been subject to UNI10772 testing with previous cameras and recently certified by the METAS institute, particularly for Swiss license plates. The collection and analysis method complied with the terms and conditions for the source of the data.

## Discussion and application

The dataset comprises 41 million passes, 14 cameras, and 4 years of observation. The raw data refers to the information collected directly from the cameras positioned throughout the Aosta Valley and can include the following details in particular:

- Timestamp of the vehicle pass: the precise time when the camera detected the vehicle, recorded with date and time (year, month, day, hour, minute, second).
- Vehicle license plate: the alphanumeric identifier of the license plate captured by the cameras. This data is in “raw” form and requires encryption for privacy reasons.
- Detection point: the specific geographical location (e.g., the entry/exit point of the Aosta Valley or the various lateral valleys) where the camera detected the vehicle.
- Direction of movement: whether the vehicle enters or exits the valley (or the specific lateral valley). The cameras can determine this based on their configuration.
- Vehicle type: some camera systems can recognize the type of vehicle (car, truck, bus, motorcycle).

The vehicle passage data analysis project in the Aosta Valley utilizes a multi-phase approach that employs advanced data management and statistical analysis techniques. The process utilizes passage data to load and manage raw data, including passage times and encrypted license plates. The license plates are encrypted to ensure privacy and contain details such as the province and the year of registration. Simultaneously, a detailed calendar of events is created. The initial DF Transit0 dataset contains vehicle passage data totaling 41,061,941 rows and 11 columns. These data form the foundation of the entire system. In parallel, a dataset related to the holiday calendar, called DF Calendar, is created, consisting of 52,610 rows and 98 columns, which includes all holidays and other relevant events that may influence traffic flows. A weather database, DF Meteo, is also created, with hourly updates, containing 43,033 rows and 673 columns, providing detailed information on weather conditions, a critical factor in traffic and tourist flow management. Subsequently, the transit data (DF Transit0) is combined with the calendar (DF Calendar) to create a new dataset called DF Transit5. This dataset has 41,061,941 rows and 148 columns and includes new variables, including the “BeforeIN” and “BeforeOUT” variables, used for imputing missing or anomalous data. At this stage, additional variables are generated to improve the quality of the analysis. Once this aggregation is complete, the data is further processed at the individual vehicle level, generating a DF Cluster dataset consisting of 5,103,316 rows and 119 columns. This dataset enables the analysis of specific vehicle behaviors, which are classified into clusters based on transit times and passage frequencies. Imputation is performed at this stage, and the data is segmented to identify homogeneous behavior groups. Starting from the DF Transit5 dataset and incorporating the cluster information, the data is further aggregated on an hourly basis. During this process, weather variables synthesized from the DF Meteo dataset are also added, forming the final analysis dataset, DF TransitA, which comprises 52,943 rows and 868 columns. This comprehensive dataset enables the application of estimation models for each checkpoint (14 in total) for different types of vehicles and drivers (7 categories) across three distinct seasons, resulting in 294 forecasting models known as EN ModelX. This modeling process is executed every 2-3 months to ensure the models remain updated and reflect the latest traffic conditions and seasonal trends. Every evening, real-time weather and new vehicle passage data are uploaded automatically. The estimated models are then used to generate updated forecasts, which are returned in the DF Predict dataset with 44,851 rows and 893 columns. Thanks to this integrated system, accurate real-time predictions of traffic and tourist flows in the region can be provided. This supports informed and strategic decision-making at both public and private levels, contributing to the optimal management of resources and infrastructure in the Aosta Valley.

## Flow records

Computational processes were performed to refine the analysis of vehicle flow, resulting in a substantial reduction of the dataset. Although corresponding to a possible loss of information, the compression allowed us to obtain a dataset focused on checkpoints and vehicle types. The meteorological data in the new dataset, called *tourist_flows.csv*, has been removed, leaving the columns that report the 14 accesses to the Aosta Valley region, categorized into the 7 types of vehicles. Although meteorological data can undoubtedly provide valuable information, especially at the predictive level, we chose not to include them in this version of the dataset. The primary objective was to develop a first forecasting framework based exclusively on the “primordial” signal of vehicle flows, to evaluate the intrinsic predictive power of traffic-based time series without the influence of external explanatory variables. This choice allowed us to obtain a cleaner benchmark and to test the robustness of the models in capturing seasonalities, trends, and structural patterns that are already embedded in the data. It is clear that weather conditions play a crucial role in shaping tourism flows, and their integration would likely enhance the accuracy of forecasts. However, introducing such additional variables would have implied a different methodological design and a broader scope. This reduced dataset establishes a solid baseline using vehicle data alone, upon which more advanced models can be built in the future. This version of the dataset presents 98 columns (30,065 rows x 99 columns, including the Date-Hour feature), which present information on the vehicle’s type by indicating the features with the letter “*c*” (see Table 2), while on the gates with the letter “*g*” (see Table 3).

**Table 2:** Vehicle type, feature descriptions of *tourist_flows.csv.*

Feature	Description
c0	Sporadic tourists
c1	Commuters
c2	Tourist (more frequent)
c3	Tourist (less frequent)
c4	Residents (more frequent)
c5	Residents (less frequent)
c6	Heavy vehicles

**Table 3:** Gates (reference road is indicated with the Italian acronyms), feature descriptions of *tourist_flows.csv.*

Feature	Reference road
g101, g102	SS26
g130, g131	A5
g241, g2061	SR45
g242, g2042	SR46
g243, g2063	SR47
g244, g2064	SR23
g300, g2060	SR44

These two types of information were combined because the features present were of the *cXgXXX* type. For example, the *c0g101* feature represented the number of sporadic tourists who passed through the initial gate of the SS26 highway (direction Aosta city). From this dataset, it was possible to obtain two sub-datasets, *cluster_g.csv*, and *cluster_c.csv*, which separate the information between access gates and vehicle type, respectively.

However, these two new datasets were constructed by adding the features with the same reference. For example, to obtain the feature *c0* of the *tourist_flows.csv* dataset, we added all the vehicles collected in the features *c0gXXX* of the starting dataset. A valid alternative to the sum could be the average between these vehicles. This operation was performed for all the features of the two sub-datasets. Finally, a time restriction was made on the days of recording the flow of vehicles. To optimize the prediction, we considered the period from 1/1/2021 to 12/31/2021 (in US format) for a total of 8,766 rows. The two datasets, following all the operations performed and cleaning, have the following dimensions (considering the Date-Hour column):

- *cluster_g.csv*: 8,766 rows x 15 columns;
- *cluster_c.csv*: 8,766 rows x 8 columns.

## Technical validation

The gates information is a time series characterized by high stationarity. As highlighted in [25], a dataset of this type (in a restricted version in terms of features) has been tested with different Machine Learning (ML) models against Deep Learning (DL) to verify which one obtains the best result in terms of prediction. Here, the dataset considered is a restriction of *cluster_g.csv*, where the feature of interest for the prediction is gate g131, while the remaining ones considered were g101, g243, g2061, and g2064. This allows us to obtain the dataset as identified in [25], consisting of 8,766 rows x 5 columns (6 columns considering the timestamp). In this case, feature selection was done manually because the corresponding geographic gates were of interest. Consequently, their geographic location prompted a “natural” feature selection. Some feature statistics are shown in Table 4.

**Table 4:** Descriptive statistics of the restricted dataset considered for the comparison between models in [25].

Feature	Mean	St. dev	Min	Max
g101	194	326	0.0	7285
g131	198	353	0.0	6750.0
g243	54	141	0.0	4241.0
g2061	69	145	0.0	2715.0
g2064	45	125	0.0	2469.0

Specifically, on the used time series which were shown to be stationary, the Machine Learning algorithms tested were eXtreme Gradient Boosting (XGBoost) [26], Random Forest [27], and *ϵ*-Support Vector Regression (*ϵ*-SVR) [28], while the Deep Learning model was a Recurrent Neural Network with Long Short-Term Memory cell (RNN-LSTM) [29]. The predictive capabilities of these were measured in terms of MAE, MSE, RMSE, and *R*^2^, highlighting how XGBoost can obtain the lowest values of MAE and MSE. Table 5 reports the main comparisons between the different models in forecasting, from [25].

**Table 5:** Comparison between Deep and Machine Learning algorithms (MAE and MSE lower the better) [25].

Model	MAE	MSE	RMSE	*R* ^2^
*XGBoost*_6_	**0.2679**	**0.3091**	**0.3559**	**0.7058**
SVR_*linear*_	0.3457	0.8211	0.9061	0.6162
SVR_*rbf*_	0.4069	1.1624	1.0781	0.4236
LSTM_2 *layers*_	0.3846	0.3368	0.5803	0.1812
LSTM_5 *layers*_	0.4311	0.3727	0.6104	0.1041
RF_10_	0.2885	0.3412	0.5841	0.6453

For each algorithm, a selection of hyperparameters was made to optimize the different models best. Specifically, as indicated in [25], the search was performed among the following values:

- XGBoost: **Max depth**=(1,6,30), **Subsample**=1, **Sampling**=Uniform, **Grow policy**=lossguide, **Learning rate**=0.3, **Lambda**=1;
- SVR: **Kernel**=(linear, rbf), **Gamma**=scale, **C**=[0.01, 5], ***ϵ***=[0.1, 1], **Coef0**=0;
- RNN-LSTM: **Layers**=[2,5], **Neurons**=[1,30], **Activation**=sigmoid, **Optimizer**=Adam, **Learning rate**=0.0005, **Batch size**=32;
- Random Forest: **Estimators**=[10,100], **Bootstrap**=True, **Oob score**=True, **Min sample split**=2.

From the perspective of the models’ characteristics, the choice of XGBoost over other models can be further justified by its algorithmic characteristics. Compared with Random Forest, which relies on bagging, XGBoost is based on gradient boosting, allowing it to reduce bias and capture more complex relationships sequentially. In addition, XGBoost incorporates advanced regularization strategies (L1/L2) and efficient handling of missing values, which contribute to improved generalization. Compared with LSTM networks, which are specifically designed to model long-term temporal dependencies, XGBoost offers a more efficient and interpretable solution in our context. In particular, LSTM models generally require larger datasets, extensive hyperparameter tuning, and higher computational costs, whereas XGBoost achieves strong performance even with moderate dataset sizes and provides more stable training.

In this case, it is possible to perform the prediction with Machine Learning (ML) algorithms that are not extremely “Deep”, considering the dataset type. Specifically, considering one gate at a time as a feature on which to perform the prediction and the others as features that influence the predictive process, the XGBoost algorithm can be used to obtain a level of prediction accuracy that is shown to be higher than Deep Learning (DL) models due to the high stationarity of the data caused by the non-constant flow of vehicles.

**Fig 2:**
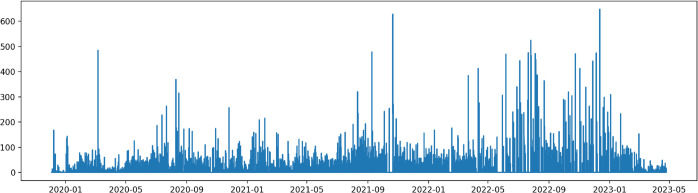
Traffic flow on the Valle de Lys gate, direction Gressoney (SR44).

For example, in Fig 2, there is a plot of the overall traffic flow recorded by the Valle de Lys gate (*g300*) for vehicles classified as tourists (*c2*). In this case, since the stationarity of the recorded data is evident even just from a graphical point of view as in [25], the XGBoost algorithm can be considered an optimal tool for predicting the future flow of vehicles. This represents a starting point for future work since an accurate predictive tool would allow regions to regulate the flow of incoming vehicles to organize the highway network better. The scalability of the structure of this dataset is very high since, for example, the entire Italian highway network uses the same (or similar models) cameras at different gates. In this way, all Italian regions could set their predictive model on their own vehicular flows. Furthermore, the applications arising from datasets like this also concern motorists. In fact, an accurate predictive model could be used by navigation systems to modify a travel route, considering, e.g., based on the date and the particular geographical area, the flow of vehicles on a certain road at a future time.

## Conclusions and limitations

This paper acknowledges some limitations related to geopolitical factors that may affect the Aosta Valley region, as well as the use of meteorological data. Vehicle flow has been employed as the primary information source, serving as a proxy for aspects such as a country’s vulnerability to financial, economic, and political risks, as well as competitiveness. These aspects, however, have not yet been translated into usable dataset features. Nevertheless, they represent a valuable starting point for future work aimed at identifying the most suitable features to capture such factors. In addition, weather data were deliberately excluded from the construction of the new datasets, to preserve the vehicle flow time series in its most original form and thus ensure a clearer characterization of its intrinsic properties. Another limitation relates to the frequency of dataset updates, which is determined by the company managing the cameras. Specifically, the company indicated that a fixed-time cycle is considered optimal, as it adequately captures seasonal variations in traffic volume. At the same time, implementing more frequent updates would be challenging due to the complexity of the acquisition process. From the perspective of applicability to other regions and future works, several improvements can be considered. First, geopolitical and socioeconomic factors could be incorporated by identifying suitable proxies and transforming them into usable features through automated feature engineering techniques. Second, meteorological data, excluded in this study, could be integrated as exogenous variables or through data fusion approaches, enhancing the robustness of traffic-related patterns. Third, regarding update frequency, alternative data sources such as IoT sensors or social media data may allow for more granular updates. Finally, the methodology could be generalized by adopting transfer learning or domain adaptation techniques, ensuring applicability even in regions where data availability and quality differ substantially.

## References

[1] Li H, Hu M, Li G. Forecasting tourism demand with multisource big data. Annals of Tourism Research. 2020;83:102912. doi: 10.1016/j.annals.2020.102912

[2] Song H, Li G. Tourism demand modelling and forecasting—a review of recent research. Tourism Management. 2008;29(2):203–20. doi: 10.1016/j.tourman.2007.07.016

[3] Lim C, McAleer M. Forecasting tourist arrivals. Annals of Tourism Research. 2001;28(4):965–77. doi: 10.1016/s0160-7383(01)00006-8

[4] Bangwayo-Skeete PF, Skeete RW. Can Google data improve the forecasting performance of tourist arrivals? Mixed-data sampling approach. Tourism Management. 2015;46:454–64. doi: 10.1016/j.tourman.2014.07.014 PMC10068136 37035094

[5] Yang J, Yang R, Chen M-H, Su C-H (Joan), Zhi Y, Xi J. Effects of rural revitalization on rural tourism. Journal of Hospitality and Tourism Management. 2021;47:35–45. doi: 10.1016/j.jhtm.2021.02.008

[6] Song C, Yang J, Wang L, Li Y, Zhi Y, Xia JC. Spatiotemporal reconstruction and drivers of tourism-oriented towns: a case study of Jinshitan. Front Environ Sci. 2022;10. doi: 10.3389/fenvs.2022.1013908

[7] Wan S, Liu L, Chen G, Wang P, Lan Y, Zhang M. Low-carbon transformation of tourism in characteristic towns under the carbon neutral goal: a three-dimensional mechanism analysis of tourists, residents, and enterprises. Sustainability. 2025;17(11):5142. doi: 10.3390/su17115142

[8] Liu Q, Zhao P, Zhang Y, Zhang Z, Yang J. Estimating the non-linear effects of urban built environment at residence and workplace on carbon dioxide emissions from commuting. Front Public Health. 2022;10:1077560. doi: 10.3389/fpubh.2022.1077560 36523576 PMC9745033

[9] Liu M, Yu Y, Zhang M, Wang P, Shi N, Ren Y, et al. Carbon balance matching relationships and spatiotemporal evolution patterns in China’s national-level metropolitan areas. Land. 2025;14(4):800. doi: 10.3390/land14040800

[10] Pu W, Quan-sheng G. An analysis of annual variation of tourist flows and climate change in Hainan Province. Geographical Research. 2009;28(4):1078–84. doi: 10.11821/yj2009040022

[11] Gössling S, Scott D, Hall CM, Ceron J-P, Dubois G. Consumer behaviour and demand response of tourists to climate change. Annals of Tourism Research. 2012;39(1):36–58. doi: 10.1016/j.annals.2011.11.002

[12] Denstadli JM, Jacobsen JKrS, Lohmann M. Tourist perceptions of summer weather in Scandinavia. Annals of Tourism Research. 2011;38(3):920–40. doi: 10.1016/j.annals.2011.01.005

[13] Lu L, Xuan G, Zhang J, Yang X, Wang D. An approach to seasonality of tourist flows between coastland resorts and mountain resorts: examples of Sanya, Beihai, Mt. Putuo, Mt. Huangshan and Mt. Jiuhua. Acta Geographica Sinica. 2002;57(6):731–40. doi: 10.11821/xb200206014

[14] Wang H, Jiang Y, Su H. Prediction of tourist flow in scenic spots based on network attention: a case study of SiGuNiang Mountain Scenic Area. In: Proceedings of the 2024 5th International Conference on Computing, Networks and Internet of Things. 2024. p. 192–7. 10.1145/3670105.3670137

[15] Fourie J, Santana-Gallego M. The impact of mega-sport events on tourist arrivals. Tourism Management. 2011;32(6):1364–70. doi: 10.1016/j.tourman.2011.01.011

[16] Song H, Witt SF. Forecasting international tourist flows to Macau. Tourism Management. 2006;27(2):214–24. doi: 10.1016/j.tourman.2004.09.004

[17] Wu DC, Song H, Shen S. New developments in tourism and hotel demand modeling and forecasting. IJCHM. 2017;29(1):507–29. doi: 10.1108/ijchm-05-2015-0249

[18] Bi J-W, Li H, Fan Z-P. Tourism demand forecasting with time series imaging: a deep learning model. Annals of Tourism Research. 2021;90:103255. doi: 10.1016/j.annals.2021.103255

[19] Hu M, Qiu RTR, Wu DC, Song H. Hierarchical pattern recognition for tourism demand forecasting. Tourism Management. 2021;84:104263. doi: 10.1016/j.tourman.2020.104263

[20] Law R, Li G, Fong DKC, Han X. Tourism demand forecasting: a deep learning approach. Annals of Tourism Research. 2019;75:410–23. doi: 10.1016/j.annals.2019.01.014

[21] Sun S, Wei Y, Tsui K-L, Wang S. Forecasting tourist arrivals with machine learning and internet search index. Tourism Management. 2019;70:1–10. doi: 10.1016/j.tourman.2018.07.010

[22] Zhang Y, Li G, Muskat B, Law R. Tourism demand forecasting: a decomposed deep learning approach. Journal of Travel Research. 2020;60(5):981–97. doi: 10.1177/0047287520919522

[23] Rakhmanov S, Habibullaev I, Jumaev A, Turgunov T. Mathematical modeling and forecasting of seasonal characteristics of tourist flow. E3S Web Conf. 2021;264:01042. doi: 10.1051/e3sconf/202126401042

[24] Zhang K, Lin Z, Zhang J. Tourist gaze through computer vision: differences between Asian, North American, and European tourists. Annals of Tourism Research. 2021;88:103039. doi: 10.1016/j.annals.2020.103039

[25] Santoro D, Ciano T, Ferrara M. A comparison between machine and deep learning models on high stationarity data. Sci Rep. 2024;14(1):19409. doi: 10.1038/s41598-024-70341-6 39169110 PMC11339414

[26] Chen T, Guestrin C. XGBoost: a scalable tree boosting system. In: Proceedings of the 22nd ACM SIGKDD International Conference on Knowledge Discovery and Data Mining. KDD ’16. New York, NY, USA: Association for Computing Machinery; 2016. p. 785–94.

[27] Breiman L. Random forests. Machine Learning. 2001;45:5–32.

[28] Sabzekar M, Hasheminejad SMH. Robust regression using support vector regressions. Chaos, Solitons & Fractals. 2021;144:110738. doi: 10.1016/j.chaos.2021.110738

[29] Hochreiter S, Schmidhuber J. Long short-term memory. Neural Comput. 1997;9(8):1735–80. doi: 10.1162/neco.1997.9.8.1735 9377276

